# Loss of zinc transporters ZIP1 and ZIP3 augments platelet reactivity in response to thrombin and accelerates thrombus formation *in vivo*

**DOI:** 10.3389/fimmu.2023.1197894

**Published:** 2023-06-08

**Authors:** Amro Elgheznawy, Patricia Öftering, Maximilian Englert, Kristina Mott, Friederike Kaiser, Charly Kusch, Uwe Gbureck, Michael R. Bösl, Harald Schulze, Bernhard Nieswandt, Timo Vögtle, Heike M. Hermanns

**Affiliations:** ^1^ Medical Clinic II, Division of Hepatology, University Hospital Würzburg, Würzburg, Germany; ^2^ Institute of Experimental Biomedicine I, University Hospital Würzburg and Rudolf Virchow Center for Integrative and Translational Bioimaging, University of Würzburg, Würzburg, Germany; ^3^ Department for Functional Materials in Medicine and Dentistry, University Hospital Würzburg, Würzburg, Germany

**Keywords:** platelets, zinc, ZIP, thrombin, signaling, thrombosis

## Abstract

Zinc (Zn^2+^) is considered as important mediator of immune cell function, thrombosis and haemostasis. However, our understanding of the transport mechanisms that regulate Zn^2+^ homeostasis in platelets is limited. Zn^2+^ transporters, ZIPs and ZnTs, are widely expressed in eukaryotic cells. Using mice globally lacking ZIP1 and ZIP3 (ZIP1/3 DKO), our aim was to explore the potential role of these Zn^2+^ transporters in maintaining platelet Zn^2+^ homeostasis and in the regulation of platelet function. While ICP-MS measurements indicated unaltered overall Zn^2+^ concentrations in platelets of ZIP1/3 DKO mice, we observed a significantly increased content of FluoZin3-stainable free Zn^2+^, which, however, appears to be released less efficiently upon thrombin-stimulated platelet activation. On the functional level, ZIP1/3 DKO platelets exhibited a hyperactive response towards threshold concentrations of G protein-coupled receptor (GPCR) agonists, while immunoreceptor tyrosine-based activation motif (ITAM)-coupled receptor agonist signalling was unaffected. This resulted in enhanced platelet aggregation towards thrombin, bigger thrombus volume under flow *ex vivo* and faster *in vivo* thrombus formation in ZIP1/3 DKO mice. Molecularly, augmented GPCR responses were accompanied by enhanced Ca^2+^ and PKC, CamKII and ERK1/2 signalling. The current study thereby identifies ZIP1 and ZIP3 as important regulators for the maintenance of platelet Zn^2+^ homeostasis and function.

## 1 Introduction

Zinc (Zn^2+^) is an essential micronutrient that represents the second most abundant transition element in the body next to iron (1, 2). In the human body, plasma contains 0.1% of total Zn^2+^, while the majority of Zn^2+^ content is present intracellularly (3, 4). Cellular Zn^2+^ exists tightly or loosely protein-bound but also in a free or non-proteinous ligand-bound status referred to as mobile or labile zinc (3). Zn^2+^ binds to nearly 10% of the human proteome to act as a cofactor for maintaining normal cellular signalling (2, 5, 6).

Zn^2+^ is present in the plasma at a concentration of 20-30 µM (with 0.5 µM in a free, non-protein bound form) and contributes to thrombosis and haemostasis by modulating platelet activation and coagulation (7–11). This is exemplified by impaired platelet reactivity and haemostatic defects in patients and animal models with dietary Zn^2+^ deficiency (12, 13). In line with this, studies have suggested that low levels (30 µM) of exogenous Zn^2+^ act as a co-factor that potentiates platelet responses to G protein-coupled receptors (GPCRs) and immunoreceptor tyrosine-based activation motif (ITAM)-coupled receptor agonists (14, 15), while intermediate to high concentrations (0.1-1 mM) of exogenous Zn^2+^ can act as platelet agonists and induce intracellular signalling, leading to granule secretion and protein kinase C (PKC)/integrin α _IIb_β_3_-dependent aggregation (14–16). Importantly, Zn^2+^ is enriched in platelets and is stored in granules or bound to metallothionein in the cytosol at concentrations 30- to 60-fold higher than in plasma (17–20). Upon platelet activation, Zn^2+^ is released into the circulation and is likely to become enriched in the microenvironment of the growing thrombus to modulate the activity of several proteins that are involved in platelet aggregation, coagulation, and fibrin clot formation (7, 8, 21–23). Consistent with that, serum has higher Zn^2+^ concentrations than plasma (24). Given the pivotal role Zn^2+^ and Zn^2+^ transporters play in infection and inflammation (25, 26), platelet-derived Zn^2+^ might be a critical contributor in immuno-thrombosis, where inflammatory processes, coagulation and platelet activation converge, e.g. in order to form thrombi to limit systemic spread of pathogens (27, 28) and also in thrombo-inflammation, in which platelets cross-talk with other immune cells to modulate the inflammatory response (29).

Since Zn^2+^ cannot permeate through the phospholipid bilayers, a regulatory machinery to actively control the transport and availability of Zn^2+^ in the cytoplasm, in different intracellular organelles, and in the extracellular space is required (8, 30). Besides a few rather unspecific channels (transient receptor potential channels TRPC6 and TRPM7 (31, 32)), Zn^2+^ homeostasis is specifically regulated by members of the *Slc30a* (zinc transporters; ZnTs) and *Slc39a* (Zrt-Irt like proteins; ZIPs) gene families (33, 34). ZIP members act as importers to mediate Zn^2+^ influx into the cytosol thereby increasing cytoplasmic Zn^2+^ levels, while ZnT isoforms work as exporters to control Zn^2+^ efflux thereby reducing cytosolic Zn^2+^ levels (35). Several ZIP and ZnT members are detected in substantial amounts in platelets and megakaryocytes (MKs) (17, 36), suggesting that Zn^2+^ homeostasis is tightly regulated in these cells. Besides Zn^2+^ transportation, also Zn^2+^ storage and buffering systems are important for the regulation of Zn^2+^ bioavailability and accumulation in response to the intracellular metabolic alterations. The best-characterized stores for free labile Zn^2+^ are the endoplasmic reticulum (ER), Golgi apparatus, vesicles and secretory granules (17, 18, 37). In addition, free mobile Zn^2+^ is sequestered by high affinity Zn^2+^-binding proteins that exist in cytosol and nucleus, in particular proteins of the metallothionein family, which bind and release Zn^2+^ atoms following alterations in the redox status (38). The mechanisms, which maintain intracellular Zn^2+^ homeostasis in platelets are unrevealed. The aim of this study is to explore the role of the two mainly plasma membrane-localized transporters ZIP1 and ZIP3 in maintaining platelet Zn^2+^ homeostasis and in the regulation of platelet function *in vivo* utilizing genetically deficient mice.

## 2 Materials and methods

### 2.1 Experimental animals

Double ZIP1/ZIP3 knockout mice (Slc39a1tm1.1Gka,Slc39a3tm1.1Gka/Mmmh) were originally described by Dufner-Beattie et al. (39). Cryopreserved spermatozoa were obtained from MMRRC, University of Missouri (RRID: MMRRC_015979-MU), and rederived by in-vitro-fertilization using C57BL/6J isogenic strain background as oocyte donor according to Takeo, T. *et* Nakagata, N. (40). Heterozygous mice were crossed to obtain wild-type and DKO litters which were further bred as homozygous lines. Mice were genotyped using the primer sets and PCR cycle parameters outlined in the genotyping protocol MMRRC 15979. Sex- and age-matched male and/or female mice were used for all experiments. Experiments were conducted according to the German animal protection law and in accordance with good animal practice as defined by the Federation of Laboratory Animal Science Associations (FELASA) and the national animal welfare body GV-SOLAS. Animal studies were approved by the district government of Lower Franconia Germany (Bezirksregierung Unterfranken, Germany).

### 2.2 *In vitro* differentiation of bone-marrow megakaryocytes

*In vitro* differentiation of MKs was carried out as described recently (41) using an antibody mixture in combination with magnetic beads for lineage depletion. The antibody negative fraction was cultured in DMEM (Gibco) containing 50 ng/ml TPO and 100 U/ml rHirudin for 72h, before the cells were enriched by straining them with a 20 µm cell strainer prior to experiments.

### 2.3 RNA isolation and quantitative RT-PCR

Total RNA was isolated from murine MKs using the NucleoSpin RNA kit (Macherey & Nagel) according to the manufacturer’s instructions including a genomic DNA digest. Reverse transcription up to 1 µg of RNA was carried out with the High-Capacity cDNA Reverse Transcription Kit (Life Technologies). Quantitative PCR (qPCR) was performed with SYBR Select Master Mix on a ViiA7 (Life Technologies). Gene expression was calculated by the comparative ΔΔCt-method and normalized to the housekeeping gene Rplp0. All primers had melting temperatures of 58-60°C (Primer Express 3.0, Life Technologies).

### 2.4 Platelet preparation

Murine blood was collected from the retroorbital plexus into 1.5 ml reaction tube containing 300 μl heparin in TBS (20 U/ml, pH 7.3). To prepare platelet-rich plasma (PRP), 200 μl TBS/heparin were added and blood was centrifuged at 800 rpm for 6 min at RT. Then, supernatant and buffy coat were transferred into a new tube containing 300 μl TBS/heparin and centrifuged at 800 rpm for 6 min at RT. To prepare washed platelets, PRP was centrifuged at 2800 rpm for 5 min at RT. The platelet pellet was gently resuspended in 1 ml Ca^2+^-free Tyrode’s buffer containing PGI_2_ (0.1 μg/ml) and apyrase (0.02 U/ml) and centrifuged at 2,800 rpm for 5 min at RT. Finally, the platelet pellet was resuspended in the appropriate volume of Ca^2+^-free Tyrode’s buffer containing apyrase (0.02 U/ml) to reach the required platelet concentration for experiments. Of note, platelet agonist concentrations might vary due to the experimental conditions.

### 2.5 Measurements of platelet cation levels using ICP-MS

Washed platelets (1x10^6^ cells/µl) were resuspended into sterile 0.9% saline solution without BSA. The cell suspension was centrifuged at 2,000 rpm for 2 min to get platelet pellets which were dissolved in 13.8% ultra-pure HNO_3_ and left for 75 min at 90°C. The lysed cells were stored at -20°C until measurements of Zn^2+^, Fe^2+^, Ca^2+^ and Mg^2+^ ion levels with ICP-MS (iCAP RQ, ThermoFisher Scientific, Waltham, USA). Before the measurements, the lysates were diluted 1:20 into ultra-pure water to be at final concentration of 0.69% HNO_3_. Five standard solutions were prepared individually for each element containing 10, 1, 0.1, 0.01 and 0.001 mg/l of Zn^2+^/Fe^2+^/Ca^2+^ ions or 100, 10, 1, 0.1 and 0.01 mg/l Mg^2+^ ions (Sigma-Aldrich, Merck KGaA, Darmstadt Germany). The blank containing 0.69% HNO_3_ was subtracted from each standard solution and sample. The internal Rh standard (Sigma-Aldrich, Merck KGaA, Darmstadt Germany) with a concentration of 10 µg/l was automatically added to each sample by the ICP-MS device. The measured ion concentration in mg/l was multiplied with 20, which means that the displayed ion concentration corresponds to the concentration in the cell lysate solution in 13.8% HNO_3_.

### 2.6 Measurement of free intracellular Zn^2+^ levels in platelets

Washed platelets (5 x 10^5^ cells/µl) were loaded with FluoZin-3 (2 µM) for 30 min at 37°C in the dark. Fluorophore-loaded platelets were washed, centrifuged at 2,000 rpm for 2 min and resuspended with Tyrode’s HEPES buffer. 25 µl of loaded platelets were diluted with 1 ml of Tyrode’s HEPES buffer. Since ZIP1/3 DKO mice express very low levels of GFP from the *Slc39a1* and *Slc39a3* gene locus (39), background mean fluorescence intensity (MFI) in non-loaded platelets was recorded for an initial 50 sec for each sample (background = F0). Then, the recording was briefly interrupted to install the tube with FluoZin-3-loaded sample derived from the same mouse, which was subsequently recorded for 50 sec (untreated = F1). The measurement was again briefly interrupted for addition of thrombin (0.02 U/ml) to the loaded platelet suspension and immediately continued for another 300 sec. At the end of this period the MFI was determined (treated = F2). Measurements were performed on a BD FACS Celesta (Becton Dickinson) and results analysed by FlowJo Software (TreeStar, USA). For generation of graphs, time series were exported to GraphPad Prism 9.5 and the respective background signal (F0) was subtracted from each sample to allow for direct comparison. The relative retained free intracellular Zn^2+^ after stimulation was determined as (F2-F0)/(F1-F0)*100 and the relative release as (F1-F2)/(F1-F0)*100.

### 2.7 Measurement of platelet dense granule release using mepacrine

50 μl of whole blood was washed twice (2,800 rpm, 5 min, RT) in Tyrode’s buffer without Ca^2+^ and finally diluted 1:10. 5 µl of washed blood was mixed with 55 µl of a 25 µM mepacrine solution and 1 µl of anti-α_IIb_β_3_-Alexa 647 antibody (clone: MWReg30 – lab made) and incubated for 30 min at 37°C. Reaction was stopped with 1.5 ml Tyrode’s without Ca^2+^. Measurements and analyses were performed analogously to FluoZin3 measurements, with gating on ${\text{α}}_{\text{IIb}}{\text{β}}_{3}^{\;+}$ cells to exclude cells other than platelets. F0 and F1 were measured for 60s, measurement time after thrombin (0.1 U/ml) addition was 180s.

### 2.8 Von Willebrand factor ELISA

Washed platelets at a concentration of 1×10^6^ platelets/μl were lysed in buffer (15 mM Tris/HCl, pH 8.0, 155 mM NaCl, 1 mM EDTA) containing 1% Igepal and protease inhibitors, incubated for 20 minutes at 4°C, and centrifuged 10 min at 14,000 rpm. For determination of Von Willebrand factor (vWF) content, 96-well plates were coated with 10 μg/ml rabbit anti-human vWF (DAKO #A0082) overnight at 4°C and blocked with 5% BSA for 2 hours at 37°C. Sample dilutions (1:200) were incubated for 2 hours at 37°C. After washing with TBS containing 0.1% Tween, samples were incubated with HRP-coupled rabbit anti-human vWF antibody (DAKO #P0226, 1:3,000) for 2 h at 37°C. After washing, ELISAs were developed using Ultra-Sensitive TMB (tetramethylbenzidine) substrate (Europa Bioproducts) and the reaction was stopped with 0.5 M H_2_SO_4_. Adsorption was determined at 450 nm and 620 nm. Background signals (wells with no sample added) were subtracted from all samples.

### 2.9 Flow cytometric analysis of platelet integrin activation, degranulation and glycoprotein expression

Measurement of platelet glycoprotein expression has been described recently (41). Briefly, 50 μl of blood were collected in 300 μl heparin in TBS and 700µl Tyrode’s buffer without Ca^2+^ was added. 50 μl of diluted blood were stained for 15 min at RT with saturating amounts of fluorophore-conjugated antibodies and analysed directly after addition of 500 μl PBS. To analyse platelet activation responses, blood samples were washed twice (2,800 rpm, 5 min, RT) in Tyrode’s buffer without Ca^2+^ and finally resuspended in Tyrode’s buffer containing 2 mM Ca^2+^. Platelets were activated with appropriately diluted agonists for 8 min at 37°C followed by 8 min at RT in the presence of saturating amounts of PE-coupled JON/A (D200, Emfret Analytics) detecting activated α_IIb_β_3_ integrin, and FITC- or DyLight649-coupled anti-P-selectin (Wug.E9, D200, Emfret Analytics and lab made) antibodies in the dark. To study the involvement of PKC, ERK1/2 or CamKII signaling pathways, platelets were pre-incubated for 1h at RT with bisindolyl-maleimide-1 (10µM), UO126 (5µM) or KN-93 (10µM), respectively. The reaction was stopped by addition of 500 μl PBS and samples were analysed with a BD FACSCelesta. Platelets were identified by their forward/side scatter (FSC/SSC) characteristics. Obtained data was analysed using FlowJo (TreeStar, USA). For FITC, background MFI of unstained platelets was subtracted.

### 2.10 Aggregometry

200 μl of washed platelets in Tyrode’s buffer containing 2 mM Ca^2+^ at a concentration of 2x10^5^ platelets/μl, were transferred into a cuvette. For all measurements with washed platelets, except those with thrombin as agonist, Tyrode’s buffer was supplemented with 100 μg/ml human fibrinogen. Agonists were added as 100-fold concentrates and light transmission was recorded over 10 min with an Apact 4-channel optical aggregation system (APACT, Hamburg, Germany). For calibration of each measurement, Tyrode’s buffer was set as 100% aggregation and washed platelet suspension was set as 0% aggregation, before the agonist was added.

### 2.11 Adhesion and spreading assay

Ibidi µ-Slide 8-well chambers were coated with 100 μg/ml human fibrinogen at 4°C overnight and blocked for at least 1h at RT with 1% BSA in sterile PBS. The wells were rinsed with Tyrode’s buffer and 300 μl washed platelets (250,000 cells/μl in Tyrode’s containing 2 mM Ca^2+^) were stimulated with thrombin (0.01 U/ml) and immediately added to the fibrinogen surface. Platelets were allowed to adhere and spread at 37°C for 10 min. The non-adhered platelets were washed away and the adhered cells were fixed by addition of 300 μl 4% PFA/PBS for 10 min at RT. Then, the wells were washed twice with 300 µl PBS. Platelets were visualized by differential interference contrast (DIC) microscopy with a Zeiss Axiovert 200 inverted microscope (100x/1.4 oil objective). Representative images were taken using a CoolSNAP-EZ camera (Visitron, Munich, Germany) and evaluated according to different platelet spreading stages with ImageJ (NIH) (42). Spreading stages were defined as follows: 1: round; no filopodia, no lamellipodia. 2: only filopodia. 3: lamellipodia; full spreading

### 2.12 Measurements of intracellular Ca^2+^ levels

Washed platelets were adjusted to a concentration of 2.5 x 10^5^/μl in Ca^2+^-free Tyrode’s buffer. 100 μl of this suspension were loaded with 3 μM fura-2-AM in the presence of 0.2 μg/mL pluronic F-127 (Life Technologies, P6867) for 20 min at 37°C. After labelling, the platelets were washed once and resuspended in 500 μl Tyrode’s buffer containing 1 mM Ca^2+^ for measurement of Ca^2+^ influx. This cell suspension was transferred into a cuvette and fluorescence was measured with a PerkinElmer LS 55 fluorimeter (Perkin Elmer, Waltham, MA, USA) under stirring conditions. Excitation was switched between 340 and 380 nm and emission was measured at 509 nm. Basal Ca^2+^ levels were recorded for 50 s before the indicated agonist was added. Each measurement was calibrated using 1% Triton X-100 and EGTA according to (43).

### 2.13 Western blotting

Washed platelets were adjusted at concentration of 4 x 10^5^/µl in BSA- and Ca^2+-^free Tyrode’s buffer and supplemented with apyrase (2 mM f.c.). Platelets were stimulated under stirring conditions in an aggregometer at 37°C, with different agonists, as indicated in the figures. The stimulation was stopped by mixing the platelet suspension with 10x concentrated ice-cold cell lysis buffer (1x f.c., Cell Signaling Technology #9803) containing Pefabloc SC-Protease-Inhibitor and phosphatase inhibitors (Carl Roth) and kept on ice for at least 30 min. Afterwards, platelet lysates were mixed with 4x SDS sample buffer (1x f.c.), boiled for 5 minutes and subjected to Western blot analysis. Samples were separated by SDS-PAGE using a 10% separating gel for electrophoresis, followed by transfer onto a PVDF membrane. Membranes were stained with Ponceau S to prove equal loading, blocked for 10 minutes in 10% BSA in TBS-T and then incubated overnight at 4°C with the indicated antibodies obtained from Cell Signaling Technology (phospho-PKC substrate motif (#6967), phospho-CamKII (#12716), phospho-p42/44 MAPK (#4370), β-actin (#3700)) or Merck/Sigma-Aldrich (tubulin (#T6074)). Afterwards, the membranes were washed three times with TBS-T at RT before being incubated with the appropriate secondary HRP-labelled antibodies (CST #7074 or #7076) for 1 h at RT. Finally, the membranes were washed several times and proteins were visualized using the Clarity™ or Clarity™ Max Western ECL Substrate (Bio-Rad) and the ChemiDoc Imaging System (Bio-Rad). For relative quantification, densitometric analyses of Western blots (non-saturated chemiluminescence and Ponceau S stainings) were carried out using ImageJ (NIH, Fiji) (42, 44) and, to allow direct comparison between blots, ratios were adjusted to one WT sample on each respective blot.

### 2.14 Collagen flow chamber assay

Coverslips (24x60 mm) were coated with 200 μg/ml fibrillar type-I collagen (Horm) overnight at 37°C and blocked for 1 h with 1% BSA at RT. Blood (700 μl) was collected into 300 μl heparin in TBS (20 U/ml, pH 7.3) and two parts of blood were diluted with one part Tyrode’s buffer with 2 mM Ca^2+^. Platelets were labelled with a DyLight-488-conjugated anti-GPIX Ig derivative (0.2 μg/ml; lab made) for 5 min at 37°C. The diluted blood was filled into a 1 ml syringe and connected to a transparent flow chamber with a slit depth of 50 μm, equipped with the coated coverslips. Perfusion was performed using a pulse-free pump under high shear stress equivalent to a wall shear rate of 1,000 s-1 for 4 min. Thereafter, coverslips were washed by a 4 min perfusion with Tyrode’s buffer at the same shear rate and phase-contrast and fluorescent images were recorded from at least five different fields of view (63x objective) using a Leica DMI6000 microscope. Image analysis was performed using ImageJ (NIH) (42, 44). Thrombus formation was expressed as the mean percentage of the total area covered by thrombi (= surface coverage) and as the mean integrated fluorescence intensity (= thrombus volume).

### 2.15 Intravital microscopy of FeCl_3_-injured mesenteric arterioles

Experiments were performed as described recently (45). Briefly, mice (16 - 19 g body weight) were anaesthetized and the mesentery was exteriorized through a midline abdominal incision. Arterioles with a diameter of 35 - 60 μm were visualized using a Zeiss Axiovert 200 inverted microscope (10x/0.25 air objective) equipped with a 100 W HBO fluorescent lamp and a CoolSNAP-EZ camera. Injury was induced by topical application of a 3 mm^2^ filter paper saturated with 20% FeCl_3_. Adhesion and aggregation of fluorescently labelled platelets (achieved by previous intravenous injection of a DyLight-488 conjugated anti-GPIX Ig derivative; lab made) in arterioles was monitored for 40 min or until occlusion occurred (blood flow stopped for >1 min). Digital images were recorded and analysed using MetaMorph software (Molecular Devices).

### 2.16 Statistical analysis

The shown data are expressed as mean ± SD or ± SEM, where appropriate. When applicable, a modified Student’s t-test was used to analyse differences between two groups. For analysis of more than two groups, two-way ANOVA for repeated measures followed by the Bonferroni multiple comparison post-hoc test was applied, p-values <0.05 were considered as statistically significant (*), p<0.01 (**) and p<0.001 (***).

## 3 Results

### 3.1 Zn^2+^ homeostasis is disturbed in platelet lacking ZIP1 and ZIP3 transporters

**Figure 1:**
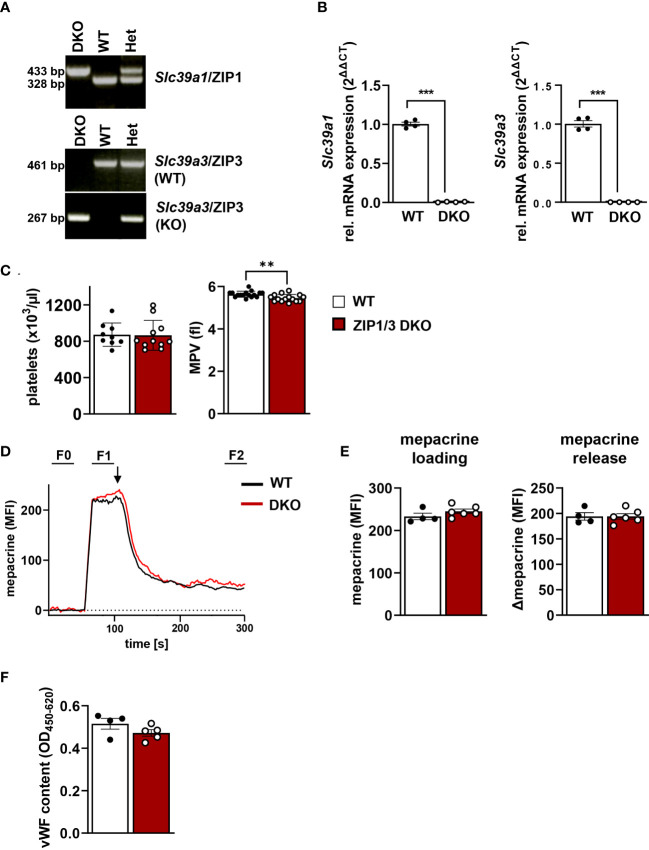
Platelets from ZIP1/3 DKO mice display a normal platelet count, but a slightly reduced platelet volume. **(A)** Representative genotyping results from genomic DNA and **(B)** quantitative RT-PCR analysis of mRNA levels of *Slc39a1* and *Slc39a3* in megakaryocytes from wild-type (WT), heterozygous (Het) and ZIP1/3 double-knockout mice (ZIP1/3 DKO), n=4. **(C)** Platelet count and volume of WT and ZIP1/3 KO mice were determined with a ScilVET analyzer, n=9-16. **(D)** Representative traces of mepacrine loading of WT and ZIP1/3 DKO platelets and stimulation with thrombin (0.1 U/ml; indicated by arrow) as measured in flow cytometry. The respective background signal (F0) was subtracted from each sample to allow for direct comparison of absolute levels and release. **(E)** Absolute mepacrine signals (MFI) after background subtraction, prior to thrombin stimulation (F1-F0) and decline of mepacrine signals upon stimulation with thrombin (F1-F2), n=4-6. **(F)** ELISA of vWF content in resting WT and ZIP1/3 DKO platelets. Data are presented as ΔOD 450 nm–OD 620 nm, n=4-5. (Student’s t-test), **P<0.01; *** P<0.001.

Several studies indicated an essential role of Zn^2+^ in the regulation of platelet activity and signalling (8–11, 14). However, the transport mechanisms that control intracellular levels of Zn^2+^ in platelets and their relevance for platelet function remained unaddressed. Our previous study indicated that several members of the ZIP (*Slc39a*) family of transporters, which regulate the import of Zn^2+^ into the cytosol (30), are expressed in MKs. RNA analysis confirmed that ZIP1 and ZIP3 were among the most highly expressed transporters in MKs during the differentiation and maturation stages (17). Therefore, we used a previously described mouse model globally lacking the expression of ZIP1 and ZIP3 transporters (ZIP1/3 DKO) (39) to study the relevance of these Zn^2+^ importers for platelet activation and signalling. Amplification of genomic DNA with primer pairs specific for either the wild-type (WT) or ZIP1/3 DKO *Slc39a1* (encoding ZIP1, **Figure 1A**, upper panel) or *Slc39a3* gene locus (encoding ZIP3, **Figure 1A**, lower two panels) confirmed the correct identity of the obtained mouse lines. Quantitative RT-PCR analyses clearly demonstrated that *in vitro* differentiated MKs of the double knock-out mice are deficient for the expression of both *Slc39a1* and *Slc39a3* (**Figure 1B**). ZIP1/3 DKO mice showed unaltered white and red blood cell counts (WBCs, RBCs: **Supplementary Figure 1**) as well as normal platelet counts (**Figure 1C**, left panel) compared to WT animals. However, there was a slight but significant reduction in platelet size in ZIP1/3 DKO mice (**Figure 1C**, right panel).

A recent report identified the zinc transporter transmembrane protein 163 (TMEM163) as critical for platelet dense granules biogenesis (20). To assess whether ZIP1/3 deficiency results in similar defects, we loaded platelets with the fluorescent dye mepacrine, which has a high affinity for adenosine nucleotides and is taken up by platelet dense granules rapidly (46). Mepacrine uptake and its release in response to a high dose of thrombin, as assessed by flow cytometry, was unaltered in ZIP1/3 DKO platelets, indicating normal dense granule content and release (**Figures 1D, E**). Likewise, the content of von Willebrand factor (vWF), a key component of platelet α-granules, was similar in lysates of WT and ZIP1/3 DKO platelets, indicative of normal α-granule content (**Figure 1F**).

**Figure 2:**
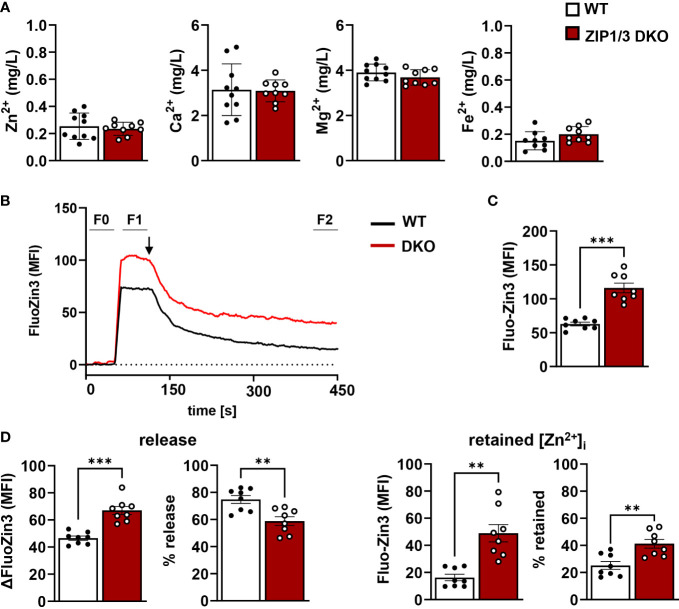
Platelets from ZIP1/3 DKO mice display increased free [Zn^2+^]_i_, but an impaired mobilisation. **(A)** Determination of intracellular amounts of Zn^2+^, Ca^2+^, Mg^2+^ and Fe^2+^ by ICP-MS analysis of washed platelets. **(B)** Representative traces of FluoZin3 labelling of WT and ZIP1/3 DKO platelets and subsequent stimulation with thrombin (0.02 U/ml; indicated by arrow). The respective background signal (F0) was subtracted from each sample to allow for direct comparison of absolute levels and release. **(C)** Absolute FluoZin3 (MFI) signals after background subtraction, prior to thrombin stimulation (F1-F0). **(D)** Released (left) and retained (right) intracellular levels of free Zn^2+^ as determined by FluoZin3 fluorescence upon stimulation of WT or ZIP1/3 DKO platelets with thrombin (0.02 U/ml, 5min). MFI and relative levels, as compared to absolute signals, are depicted. n= 8-10, (Student’s t-test), **P<0.01; *** P<0.001.

Since ZIP1 and ZIP3 act as importers to promote Zn^2+^ influx into the cytosol, we determined total levels of intracellular Zn^2+^ (protein-bound and labile) by inductively coupled plasma mass spectrometry (ICP-MS). No significant differences were found for overall [Zn^2+^]_i_ or other divalent cations ([Ca^2+^]_i_, [Mg^2+^]_i_, [Fe^2+^]_i_) (**Figure 2A**). To determine the relative ratio of free intracellular Zn^2+^, WT and ZIP1/3 DKO washed platelets were incubated with the Zn^2+^-specific cell permeable fluorescent dye Fluo-Zin3 (**Figure 2B**). Platelets from DKO mice contained significantly more FluoZin3-stainable [Zn^2+^]_i_ than WT controls (**Figure 2C**), which, however, was not as efficiently released from the cells in response to thrombin (0.02 U/ml) as in WT platelets (**Figure 2D**, left panels): while WT platelets retained only 25.21% ± 2.75 (n=8) of the background-corrected FluoZin3 fluorescence signal, ZIP1/3 DKO platelets retained 41.19% ± 3.03 (n=8; p<0.01, **Figure 2D**, right panels). Because of the higher FluoZin3-stainable [Zn^2+^]_i_ content, the total amount of Zn^2+^ released by the DKO platelets was nonetheless increased as compared to WT, despite the mobilization defects (**Figure 2D**, left panels).

### 3.2 Platelets lacking ZIP1 and ZIP3 transporters are hyperreactive to threshold concentrations of GPCR agonists

**Figure 3:**
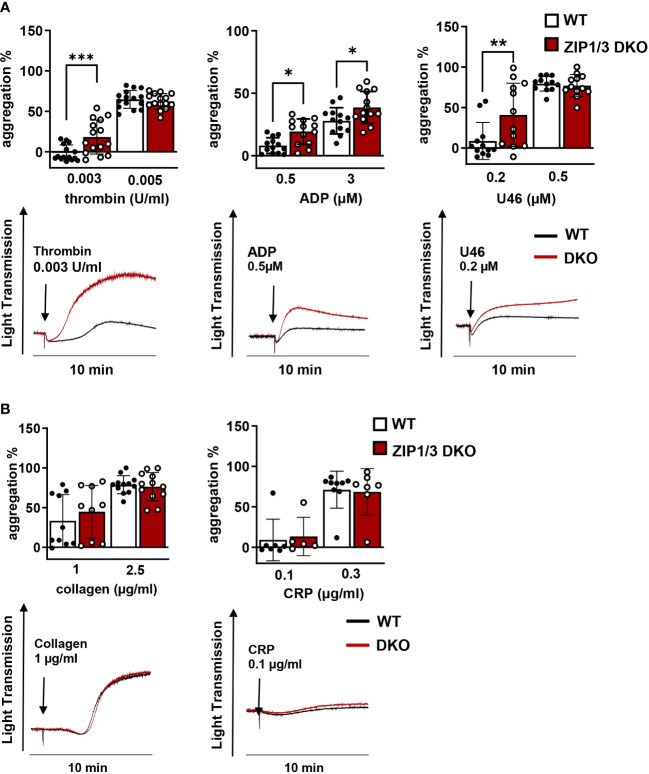
Platelets from ZIP1/3 DKO mice are hyperresponsive towards GPCR agonists, but not to ITAM-coupled receptor agonists in aggregometry. Platelet aggregation was measured using Fibrintimer 4-channel aggregometer after stimulation of washed platelets isolated from WT and ZIP1/3 DKO mice with **(A)** different GPCR agonists (thrombin, ADP, U46619) and **(B)** ITAM-coupled receptor agonists (collagen, CRP) using the indicated concentrations; n=5-15 (two-way ANOVA + Bonferroni), *P<0.05, **P<0.01,***P<0.001.

Activated platelets release Zn^2+^ into the circulation to modulate proteins that play a major role in platelet adhesion and aggregation (7, 21–23). Furthermore, previous studies have indicated that Zn^2+^ can act as agonist or second messenger to modulate platelet reactivity in a concentration-dependent manner (10, 14). With respect to the observed altered [Zn^2+^]_i_ in ZIP1/3 DKO mice (**Figures 2B–D**) and previous findings that disturbed [Zn^2+^]_i_ alters platelet function, we evaluated the aggregation response of WT and ZIP1/3 DKO platelets. Interestingly, ZIP1/3 DKO mice displayed an enhanced platelet aggregation in response to threshold concentrations of GPCR agonists such as thrombin (0.003 U/ml), ADP (0.5 µM) and the thromboxane A2 (TxA_2_) analogue U46619 (0.2 µM) (**Figure 3A**). However, in response to threshold concentrations of collagen (1 or 2.5 µg/ml) or collagen-related peptide (CRP, 0.1 or 0.3 µg/ml) (**Figure 3B**), agonists of the ITAM-coupled receptor glycoprotein (GP)VI, there was no significant difference in platelet aggregation between ZIP1/3 DKO and their corresponding WT controls. This suggested that loss of ZIP1/3 might alter GPCR-coupled signalling in activated platelets.

**Figure 4:**
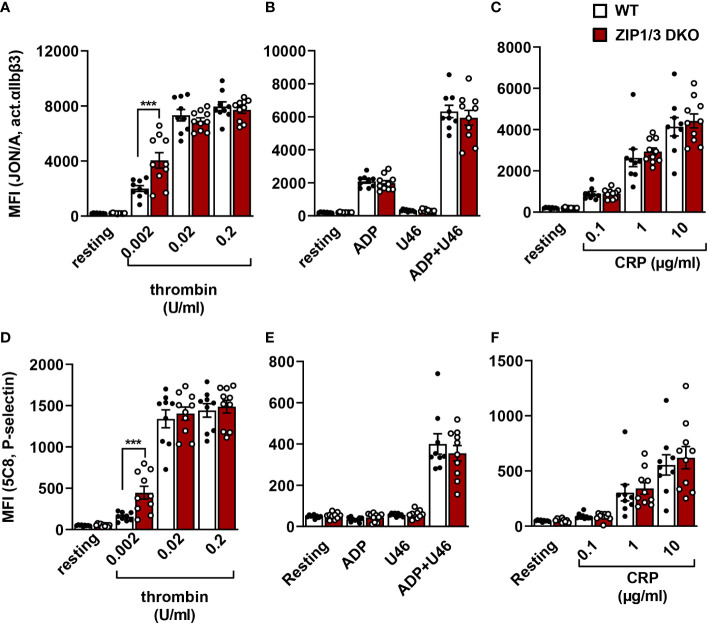
Analysis of platelet integrin activation and degranulation by flow cytometry. Platelets from WT and ZIP1/3 DKO mice were activated with either thrombin **(A, D)**, ADP (10µM), U46619 (3µM) and their combination **(B, E)** or CRP **(C, F)** and stained with saturating amounts of PE-coupled JON/A detecting activated αIIbβ3 integrin **(A–C)** or FITC-coupled anti-P-selectin **(D–F)**. Mean background fluorescence of unstained platelets was subtracted from all data sets. n=9-10 (two-way ANOVA + Bonferroni), ***P<0.001.

To corroborate this hypothesis, we measured the inside-out activation of the major platelet integrin α_IIb_β_3_ (vWF/fibrinogen receptor) in flow cytometry using the JON-A/PE antibody, which binds α_IIb_β_3_ only in its active conformation (**Figures 4A–C**). Our analysis showed that ZIP1/3 deficiency promoted α_IIb_β_3_ integrin activation in response to threshold concentrations of thrombin (0.002 U/ml) (**Figure 4A**), however, responses to ADP, U46619 (**Figure 4B**) or the ITAM receptor agonist CRP (**Figure 4C**) remained overall unaffected. Similar observations were made for the degranulation-dependent P-selectin surface exposure (**Figures 4D–F**). Of note, there was no difference in the total expression levels of integrins (α_IIb_β_3_, α2 and α5) in platelets from ZIP1/3 DKO and WT mice (**Supplementary Figure 2A**). Similarly, no differences were detected in the surface expression levels of the GPIb-V-IX complex (**Supplementary Figure 2B**), CD9, CLEC2 and GPVI (**Supplementary Figure 2C**).

**Figure 5:**
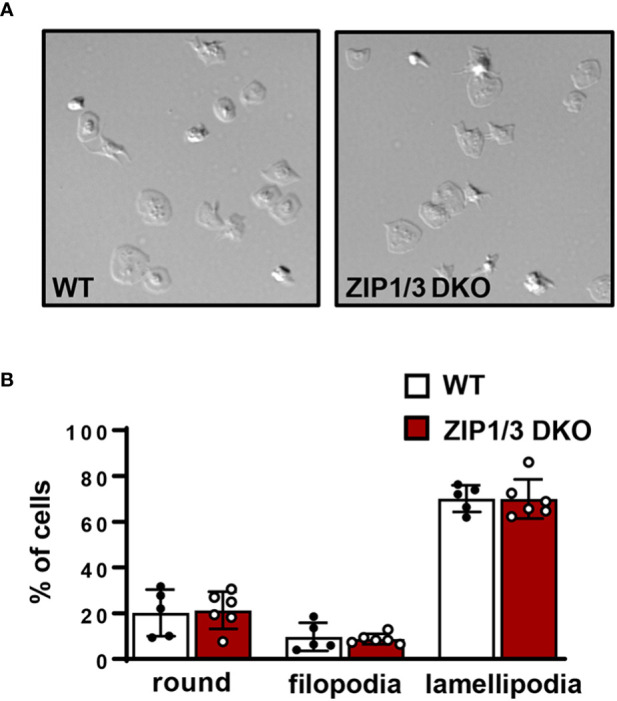
ZIP1/3 deficiency has no effect on *in vitro* platelet adhesion and spreading under static conditions. **(A)** Washed platelets from WT and ZIP1/3 DKO mice were allowed to adhere on fibrinogen for 10 min in the presence of 0.01 U/ml thrombin. **(B)** Quantification of spreading stages (round=no filopodia, no lamellipodia). n=5-6, (Student’s t-test).

Since it has been reported that intracellular Zn^2+^ concentrations affect platelet shape change, indicative for altered cytoskeletal dynamics (10), we tested platelet adhesion and spreading on fibrinogen in the presence of thrombin under static conditions. However, platelets isolated from ZIP1/3 DKO mice did not show any significant difference in adhesion and spreading compared to WT mice (**Figures 5A, B**).

### 3.3 ZIP1/3 deficiency increases Ca²^+^ influx and PKC activation in platelets

**Figure 6:**
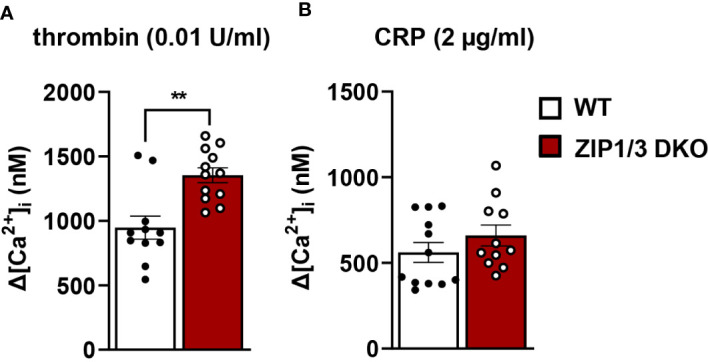
ZIP1/3 deficiency increases Ca²^+^ influx in response to thrombin but not in response to CRP. The increase in intracellular Ca^2+^ levels ([Ca^2+^]_i_) in washed platelets from WT and ZIP1/3 DKO mice stained with Fura-2 A/M after the stimulation with **(A)** thrombin (0.01 U/ml) or **(B)** CRP (2 µg/ml) in the presence of 1 mM CaCl_2_; n=12, (Student’s t-test), **P<0.01.

**Figure 7:**
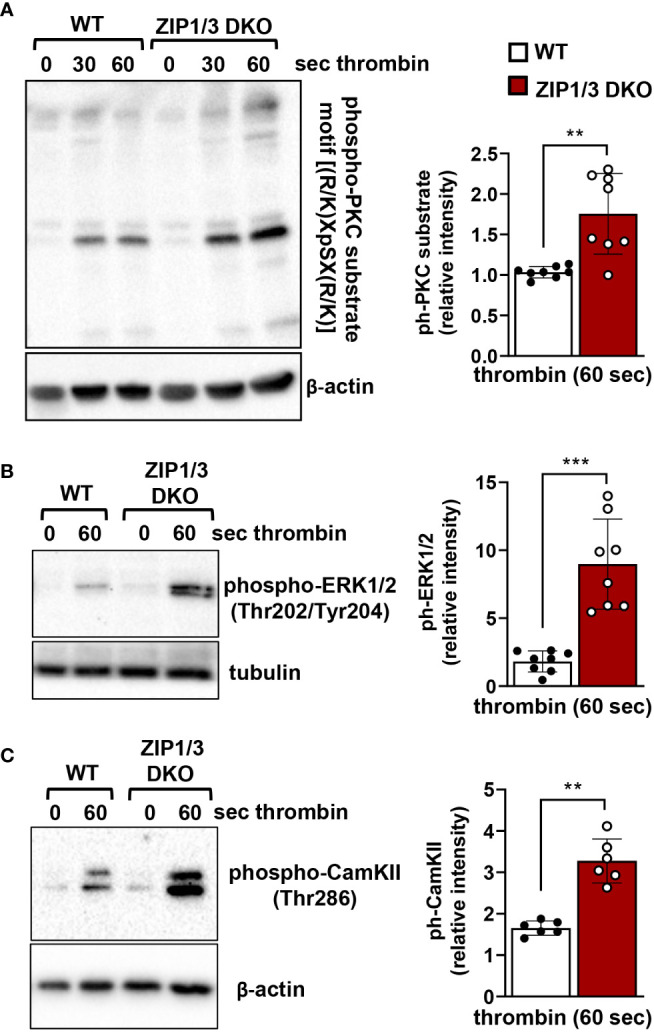
ZIP1/3 deficiency promotes PKC, ERK1/2 and CamKII activation in murine platelets. Left: Representative blots show the levels of the phosphorylation of PKC substrates **(A)**, ERK1/2 **(B)** and CamKII **(C)** under resting (0s) conditions or upon stimulation with thrombin (0.003 U/ml for 30s and/or 60s). Staining against β-actin or tubulin served as loading control. Right: Densitometric analysis of signals obtained for the respective phosphorylated proteins normalized to loading control; n=6-8, (Student’s t-test), **P<0.01, ***P<0.001.

Recent work suggests that Zn^2+^ may work synergistically with Ca^2+^ to induce platelet activation (14). Even though overall [Ca^2+^]_i_ appeared to be equivalent in WT and ZIP1/3 DKO platelets at resting conditions in the absence of extracellular Ca^2+^ as determined by ICP-MS (**Figure 2A**), Ca^2+^ mobilisation was significantly enhanced in ZIP1/3 DKO mice in response to the GPCR agonist thrombin (0.01 U/ml) (**Figure 6A**), while Ca^2+^ mobilisation in response to the GPVI agonist CRP was not significantly altered (**Figure 6B**). Given the increase in Ca^2+^ mobilisation in response to thrombin (**Figure 6A**) and the fact that increased [Zn^2+^]_i_ has been shown to modulate protein kinase C (PKC) (14), we assessed the importance of platelet ZIP1/ZIP3 in agonist-mediated activation of PKC by determination of the phosphorylation status of PKC substrates using Western blot analyses. Deletion of ZIP1/3 resulted in elevated phosphorylation levels of PKC substrates in response to low-dose thrombin (0.003 U/ml) (**Figure 7A**) compared to platelets from WT mice. Interestingly, also the extracellular signal-regulated kinases 1 and 2 (ERK1/2) (**Figure 7B**) and the Ca^2+^/calmodulin-dependent kinase II (CamKII) (**Figure 7C**) displayed an increase in phosphorylation status in ZIP1/3 DKO platelets. Incubation of platelets with inhibitors against PKC (bisindolylmaleimide-1), MEK1/2 (UO126) or CamKII (KN-93) completely blocked low-dose thrombin dependent exposure of P-selectin and α_IIb_β_3_ activation (**Supplementary Figure 3**). Therefore, the absence of ZIP1 and ZIP3 transporters resulted in an increase in platelets’ sensitivity to Ca^2+^-dependent thrombin signalling.

### 3.4 Deficiency of ZIP1/3 transporters promotes thrombus formation

**Figure 8:**
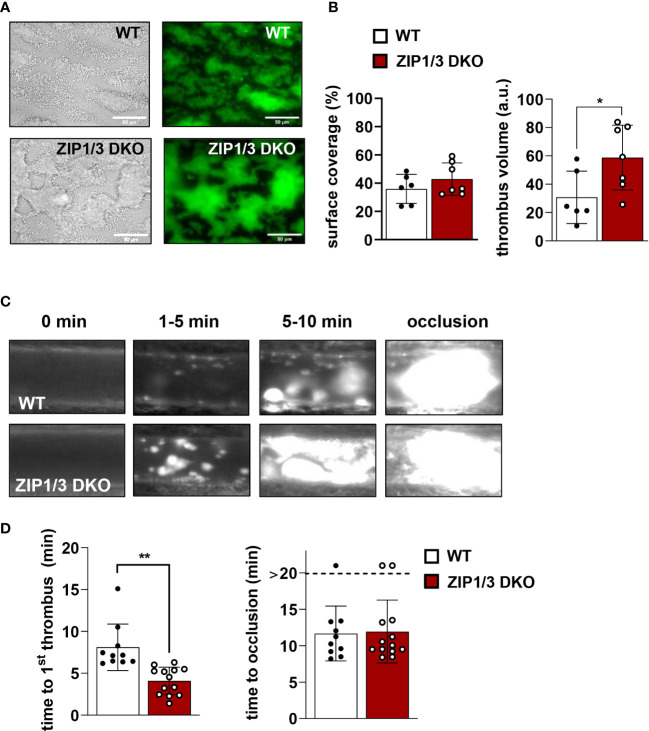
ZIP1/3 deficiency promotes *ex vivo* and *in vivo* thrombus formation. Adhesion and thrombus formation of platelets on collagen was assessed in a flow adhesion assay at a wall shear rate of 1000/s. Representative images **(A)** and the quantification **(B)** of the surface coverage and the relative thrombus volume are shown (n = 6-7; Student’s t-test; *P<0.05; Scale bar: 50 μm). Thrombus formation upon FeCl_3_-mediated injury of mesenteric arteries. Representative images **(C)** and quantitative analysis **(D)** indicate the time for appearance of 1^st^ *in vivo* thrombus (> 10 µM) and time to occlusion. Each dot represents one mesenteric artery, n=10-13, (Student’s t-test); *P<0.05, **P<0.01.

Zn^2+^ is considered to be an important contributor to haemostasis and thrombosis (8). According to this information and with respect to the observed hyperreactivity in thrombin-, ADP- and U46619-mediated aggregation (**Figure 3A**), we determined the thrombotic potential in WT and ZIP1/3 DKO mice *ex vivo* and *in vivo*. In an *ex vivo* flow chamber assay, in which whole blood was perfused over collagen-coated coverslips at a shear rate of 1000s^-1^, we observed significantly bigger stable 3-dimensional platelet aggregates in case of blood from ZIP1/3 DKO mice, while surface coverage was only mildly affected (**Figures 8A, B**). Consistent with these *ex vivo* findings, ZIP1/3 DKO mice formed clearly visible thrombi at earlier time points *in vivo* upon FeCl_3_-mediated injury of mesenteric arteries. However, the time to occlusion did not differ significantly between ZIP1/3 DKO and WT mice (**Figures 8C, D**).

## 4 Discussion

Zn^2+^ homeostasis is mainly controlled through Zn^2+^-transporting and -binding proteins. Transporters of the ZIP superfamily are widely expressed in eukaryotic cells to import Zn^2+^ into the cytoplasm, while ZnT proteins export Zn^2+^ out of the cytoplasm (33, 34). Insofar as ZIPs and ZnTs are transmembrane proteins, they are located in the plasma membrane, the ER/Golgi network membranes as well as in intracellular vesicles/granules to coordinate Zn^2+^ transport. Most data available so far on their exact localisation originate from experiments with transfected cells expressing tagged proteins, which allow utilisation of well-established antibodies for detection (47–49). Many commercially available antibodies to ZIPs appear to display cross-reactivity with other family members and to the best of our knowledge comparative data for antibodies utilizing knock-out or knock-down cells are missing and urgently required. This might explain why the role and localisation of endogenous ZIP transporters in platelets, as in other cell types, remains difficult to address up to date. The global deletion of ZIP1 and ZIP3 provided the first direct proof that ZIP transporters are involved in the regulation of Zn^2+^ homeostasis in hippocampal neurons (50). Our presented study is, to our knowledge, the first to investigate the importance of individual ZIP transporters for platelet Zn^2+^ homeostasis and activation. Utilizing mice, which globally lack ZIP1 and ZIP3, we provide evidence that these transporters are involved in platelet Zn^2+^ homeostasis and contribute to the control of platelet sensitivity towards GPCR agonists, in particular thrombin, without affecting ITAM receptor agonists. Since ZIP1/3 DKO mice did not display alterations in platelet count, we conclude that megakaryopoiesis is independent of the presence of these transporters despite a significant increase in *Slc39a1* (ZIP1) transcription during *in vitro* differentiation of MKs from haematopoietic stem cells (HSCs) (data not shown). Similarly, we did not observe any defects in other blood cells which is in line with the initial description of the ZIP1/3 DKO mouse line (39). Due to the lack of specificity of all tested commercially available antibodies to detect ZIP1 and ZIP3, we confirmed the deletion of ZIP1 and ZIP3 by qPCR analysis in MKs generated *in vitro* from HSCs. Platelets from ZIP1/3 DKO mice showed increased levels of free intracellular Zn^2+^, despite their deficiency in two Zn^2+^ import transporters. Because of the global lack of ZIP1 and ZIP3, we cannot exclude an indirect increase in platelet [Zn^2+^]_i_, e.g. due to buffering elevated plasma Zn^2+^ levels that might originate from a lack of Zn^2+^ uptake into other cell types. However, data from ZIP1/2/3 triple KO mice show unaltered uptake of ^67^Zn into the liver under a Zn-adequate feeding regime (51). Therefore, strongly altered Zn^2+^ plasma levels appear unlikely as this should result in altered uptake into hepatocytes as well (52). As further possible explanation, other ZIPs might be up- or ZnTs downregulated in a compensatory mechanism. Our previous studies identified substantial amounts of *Slc39a4* (ZIP4), *Slc39a6* (ZIP6), *Slc39a7* (ZIP7), *Slc39a9* (ZIP9), *Slc39a10* (ZIP10) and *Slc30a1* (ZnT1) mRNA in MKs (17). At least for *Slc39a4*, *Slc39a10* and *Slc30a1* we did not detect a compensatory altered transcription in MKs from ZIP1/3 DKO mice (data not shown). Since FluoZin3-stainable free Zn^2+^ appears to be largely sequestered in granules (17, 20), it might also be possible that ZIP1/3 are located not only at the plasma membrane in platelets, but also in the membrane of granules to regulate together with ZnTs the exchange of free Zn^2+^ between granules and cytoplasm. Protein expression or cellular localisation of the transporters might be changed in MKs and platelets of ZIP1/3 DKO mice, however, as mentioned above due to the lack of available specific antibodies we were unable to address this hypothesis in the present study.

The relative release of free Zn^2+^ in response to thrombin was less efficient in ZIP1/3 DKO as compared to WT platelets, resulting in elevated levels of [Zn^2+^]_i_ post activation. As mentioned above, potential mechanisms might involve a dysregulated expression, localisation or activity of ZIP/ZnT transporters or alternatively a defective release of granules. A general defect in α- or dense granule biogenesis and release, however, can be excluded since the content of vWF factor as well as the uptake/release of mepacrine is unaltered and the exposure of P-selectin is rather increased in response to thrombin in ZIP1/3-deficient platelets. Interestingly, recent work indicated that α-granules might not represent a homogeneous population of organelles, but rather comprise a group of subcellular compartments with unique composition and ultrastructure, which might even release their content in an ordered manner (53–55). Whether ZIP1/3 may contribute to a selective release of platelet granules needs to be addressed in future experiments. Alternatively, Zn^2+^ might be stored in organelles or the cytoplasm in a way that cannot be mobilized upon activation. Indeed, the appearance of the FluoZin3 uptake and release curves (**Figure 2B**) suggest that the excess of free Zn^2+^ in ZIP1/3 DKO platelets might reside in such a non-releasable intracellular pool.

So far, no data were available about the mechanisms by which Zn^2+^ transporters control platelet activity. The observed hyperresponsiveness of platelets lacking ZIP1 and ZIP3 might be attributed to the increased retained pool of free intracellular Zn^2+^ which can act as a second messenger to enhance phosphorylation events as shown in platelets (10, 11, 14), but also in nucleated cells, in particular mast cells (56), or might directly impact redox signalling (57). In addition, elevated [Zn^2+^]_i_ might interfere with the intracellular Ca^2+^ homeostasis and a number of recent studies suggest an elaborate cross-talk between these cations (14, 58–60). Interestingly, we observe an enhanced Ca²^+^ mobilisation in response to thrombin, but not CRP, in ZIP1/3 DKO platelets, which likely affects a number or downstream signalling pathways. Of note, Fura-2 which is the commonly utilized fluorophore to determine Ca^2+^ mobilisation in cells and which has also been used in our experiment, has been shown to additionally bind Zn^2+^. However, we consider it unlikely that the increased Fura-2 fluorescence signal observed upon platelet activation with thrombin (as shown in **Figure 6A**) is due to the elevated [Zn^2+^]_i_, since the free intracellular concentrations between both divalent cations differ massively (Zn^2+^: 0.4 nM; Ca^2+^: 100 nM (60)). The determined thrombin-dependent Ca^2+^ mobilisation (extracellular influx and intracellular store release) was calculated to result in 1300 nM [Ca^2+^]_i,_ which makes a significant unspecific detection of Zn^2+^ by Fura-2 in this context very unlikely.

A common signalling molecule on which Zn^2+^ and Ca^2+^ pathways converge is PKC. It has been reported that Zn^2+^ augments PKC activity in lymphocytes and platelets (16, 61). Besides Zn^2+^, Ca^2+^ activates PKC to regulate PS exposure in platelets (62). We observed an increased activation of PKC in ZIP1/3-deficient platelets in response to thrombin as demonstrated by the enhanced phosphorylation of proteins harbouring the PKC substrate motif. Taken into consideration that PKC is essential for thrombus formation (63) (**Supplementary Figure 3**), enhanced PKC activation could explain the observed accelerated thrombus formation in ZIP1/3 DKO mice. Given that PKC activation mediates platelet granule secretion (63, 64) and positively regulates integrin activation (65), the data are consistent with the increase of P-selectin exposure and α_IIb_β_3_ activation in platelets from ZIP1/3 DKO mice in response to thrombin. Downstream of PKC, activation of ERK1/2 has been described for platelet activation. Indeed, contrary to most other cell types, in platelets PKC rather than Raf isoforms represents the major MAP3K, particularly in response to thrombin stimulation (66–68). Consistent with the increased activity in PKC, we observe enhanced phosphorylation of ERK1/2 on Thr202/Tyr204 in the activation loop of the kinases. Increased activation of ERK1/2 could well contribute to the hyperresponsiveness of ZIP1/3 DKO platelets since numerous groups and our own data show defects in platelet adhesion as well as α_IIb_β_3_ activation and platelet aggregation upon inhibition of these kinases by pharmacological inhibitors (69). Notably, we have also observed an increased phosphorylation of CamKII at Thr286 in response to low dose thrombin, which correlates with the kinase’s activity (70). Consistent with this observation, a study in neurons reported modulation of CamKII activity by Zn^2+^ (71). Activated CamKII has been shown to enhance store-operated Ca^2+^ entry (SOCE) in heterologous cell systems by promoting the interaction of STIM1 and Orai1 in a positive feedback loop (72). Since STIM1/Orai1-mediated SOCE is the main Ca^2+^ entry mechanism following agonist activation in platelets (73), the enhanced CamKII activation therefore might contribute to the observed enhanced Ca^2+^ influx in response to thrombin and thereby to platelet activation. Taken together, our findings in platelets from genetically modified ZIP1/3 DKO mice confirm for the first time *in vitro* results of other groups, in which different concentrations of Zn^2+^ or the Zn^2+^ ionophores clioquinol/pyrithione were applied exogenously to platelets and promoted platelet activation as well as PKC or ERK1/2 activation (10, 11, 57).

In aggregometry experiments ZIP1/3-deficient platelets were hyperresponsive in response to threshold concentrations of GPCR agonists, but not towards agonists of the ITAM-coupled receptor GPVI, namely CRP and collagen. Nevertheless, platelets from ZIP1/3 DKO formed larger thrombi when perfused over a collagen-coated surface. The observed differences in these assays might be consequences of the different experimental setups. In aggregometry, CRP and collagen are in solution and thus able to activate all platelets in the sample during the activation period. Hence, due to the release of second wave mediators we observe synergistic GPVI/GPCR effects. In contrast, in flow chamber experiments, collagen is mainly interacting with platelets that are in direct contact with the flow cell surface. This interaction is measured as surface coverage and unaffected in ZIP1/3 DKO platelets. The subsequent build-up of a three dimensional thrombus on top of this layer is facilitated by platelets that are not in direct contact with the collagen-coated surface, but are activated by second wave mediators, such as TxA_2_ and ADP, which signal *via* GPCRs. Indeed, previous studies showed that co-infusion of ADP and the stable TxA_2_ analogue U46619 into the flow chamber could enhance thrombus formation (74), hence arguing that the observed phenotype is due to enhanced GPCR signalling in ZIP1/3 DKO platelets. Interestingly, one of the identified substrates of ERK1/2 activity in platelets is cytosolic phospholipase A_2_ (cPLA_2_) and MEK1/2 inhibitors attenuated agonist-induced cPLA_2_ phosphorylation and downstream thereof TxA_2_ secretion in stimulated platelets (75, 76). GPCR-mediated signalling pathways are also prevailing in our model of FeCl_3_-induced vessel injury (77–79), which is triggered by oxidative damage of vessel wall and blood cells and flocculation of blood proteins, while there is only limited, if any, collagen exposure inside the vessel (78, 80, 81). Therefore, the accelerated thrombus formation in ZIP1/3 DKO mice is again in line with the hyperresponsiveness of their platelets towards lower doses of GPCR agonists. Of note, since a global KO model was used, we cannot exclude an impact of other cell types, e.g. endothelial cells and blood cells, as well as systemic alterations on the experimental outcome.

Besides these platelet intrinsic contributions of Zn^2+^ signalling to thrombosis, a further aspect that warrants discussion is the role of platelet-released Zn^2+^ in modulating immune cell activation and the contact activation system in the processes of immunothrombosis and thrombo-inflammation. Platelet-neutrophil interactions have been observed in a plethora of diseases and are known to promote the release of neutrophil extracellular traps (NETs), a hallmark of immunothrombosis (27, 82). Although the exact effects are controversially discussed, several studies have shown that both Zn^2+^ overload and Zn^2+^ deficiency modulate NET formation (83), implying that microenvironmental changes of Zn^2+^ concentrations by release from platelets might modulate this process. One consequence of NET formation is the activation of FXII, which can trigger the contact activation pathway and the kinin-kallikrein pathway and is suspected to contribute to the pathogenic chain of procoagulant and proinflammatory responses in COVID-19 (84). Since Zn^2+^ is required for autoactivation of FXII, and because platelets are able to modulate FXII function in a Zn^2+^-dependent manner (23, 85), it will be interesting to investigate how alterations in platelet Zn^2+^ release and alterations in the microenvironment impact FXII activation in future studies.

Taken together, our study for the first time attributes an important regulatory role to zinc transporters in platelet activation. *In vivo*, ZIP1/3 DKO mice develop faster thrombi in response to vascular injury. *In vitro* data show that platelets from ZIP1/3-deficient mice are hyperreactive to threshold concentrations of GPCR agonists, in particular thrombin, while responses to ITAM-coupled receptor agonists appear to be unaffected. Thereby our data encourage future studies to delineate the possible exclusive involvement of individual Zn^2+^ transporter in platelet activation in response to different agonists and their role in thrombosis. This holds particularly true for situations that require a balanced contribution of platelets to the immune response as in immunothrombosis or thrombo-inflammation.

## Data availability statement

The original contributions presented in the study are included in the article/**Supplementary Material**. Further inquiries can be directed to the corresponding authors.

## Ethics statement

The animal study was reviewed and approved by Regierung von Unterfranken.

## Author contributions

AE, PÖ, ME, KM, FK, CK, UG, TV and HH performed experiments and analysed data. HS analysed data and together with BN discussed results, provided scientific input and technical equipment throughout the study. MRB generated the mice. AE, TV, HH wrote the manuscript with input from all the authors. AE, TV and HH conceived the study and designed research. All authors contributed to the article and approved the submitted version.
