# Supplementary material for: Loss of zinc transporters ZIP1 and ZIP3 augments platelet reactivity in response to thrombin and accelerates thrombus formation in vivo
Source: Front Immunol. 2023 Jun 8;14:1197894. doi: 10.3389/fimmu.2023.1197894 (PMC10285393; doi:10.3389/fimmu.2023.1197894)
Supplement: Supplementary file 1 [file DataSheet_1.pdf]

Supplementary Material

Loss of zinc transporters ZIP1 and ZIP3 augments platelet reactivity in response to thrombin and accelerates thrombus formation *in vivo*

Elgheznawy A, Öftering P, Englert M, Mott K, Kaiser F, Kusch C, Gbureck U, Bösl MR, Schulze H, Nieswandt B, Vögtle T, Hermanns HM*

*** Correspondence:** Hermanns_H@ukw.de, Voegtle_T@ukw.de

1 Supplementary Data

No supplementary data

2 Supplementary Figures and Tables

2.1 Supplementary Figures

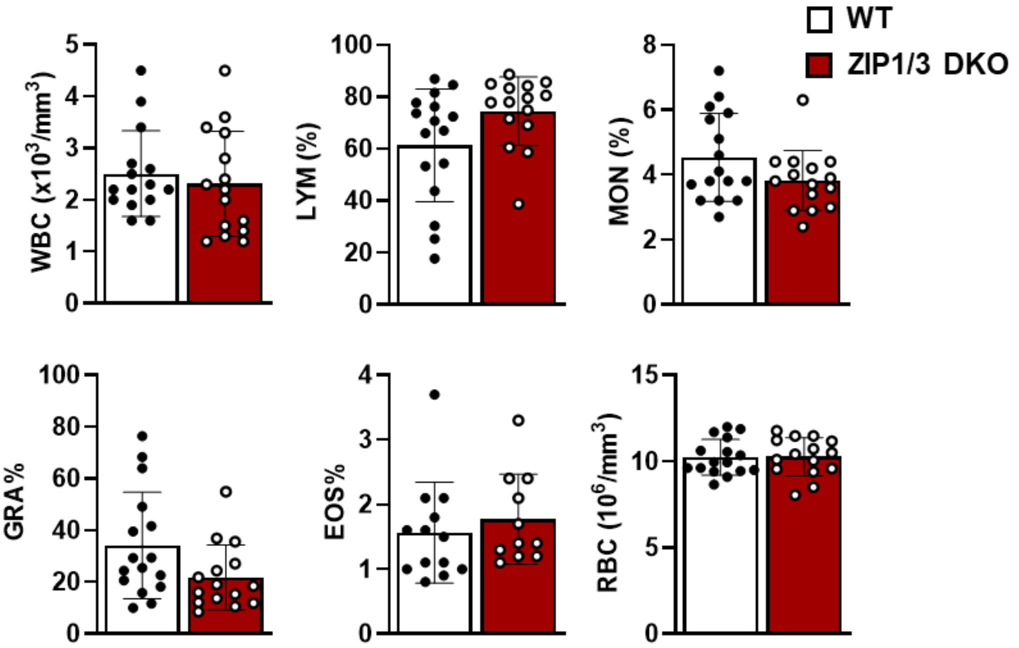

Supplementary Figure 1. ZIP1/3 deficiency has no effect on white and red blood cell count. Analyses of blood parameters were performed from EDTA blood using a ScilVET hemacytometer. n=15-16 (Students t-test).

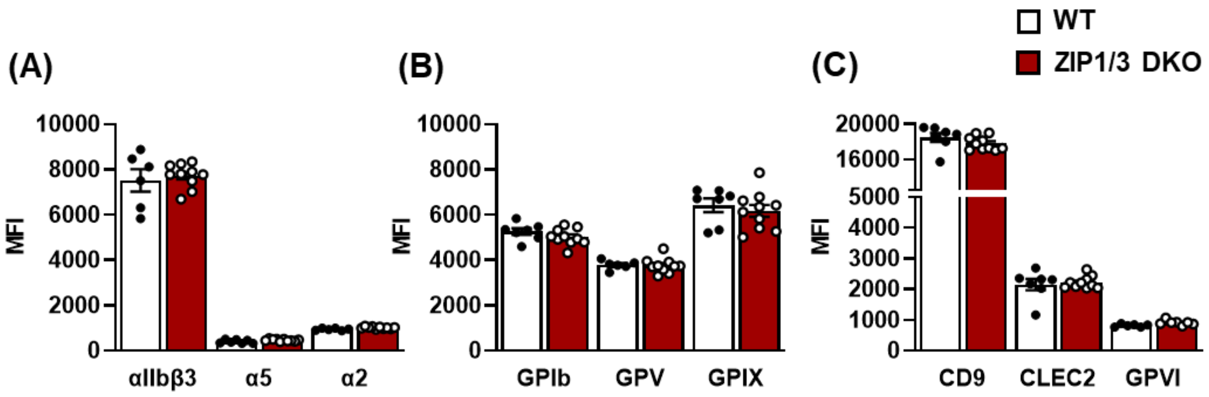

Supplementary Figure 2. ZIP1/3 deficiency does not affect integrin or platelet glycoprotein cell surface levels. Flow cytometric analysis of the cell surface expression of the indicated integrins (A), the GPIb-V-IX complex (B) and CD9, CLEC2, GPVI (C) using directly fluorescently-labelled specific antibodies in saturating amounts. n=6-10 (Students t-test).

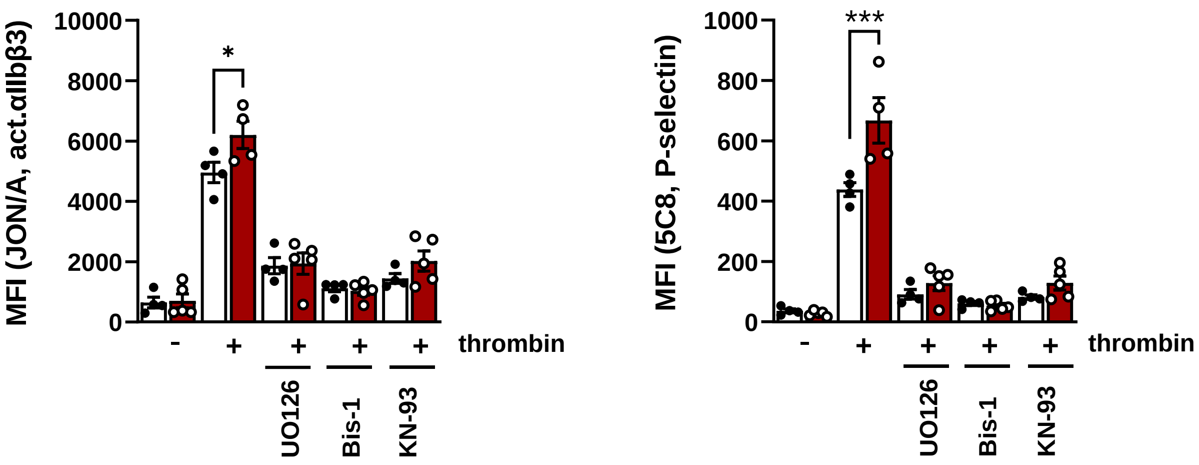

Supplementary Figure 3: Influence of PKC, ERK1/2 and CaMKII inhibitors on thrombin-stimulated integrin activation and degranulation. Platelets from WT and ZIP1/3 DKO mice were pre-incubated for 1h at RT with inhibitors against PKC (bisindolylmaleimide-1 (Bis-1); 10 µM), MEK1/2 (UO126; 5µM) or CamKII (KN-93; 10 µM), activated with a threshold dose of thrombin and stained with saturating amounts of PE-coupled JON/A detecting activated $\alpha_{IIb}\beta_3$ integrin (left) and Dylight649-coupled anti-P-selectin (right); n=4-5 (two-way ANOVA + Bonferroni; *P<0.05, ***P<0.001).
